# Supplementary material for: Effects of Lactobacillus plantarum and Weissella viridescens on the Gut Microbiota and Serum Metabolites of Mice with Antibiotic-Associated Diarrhea
Source: Nutrients. 2023 Oct 30;15(21):4603. doi: 10.3390/nu15214603 (PMC10648191; doi:10.3390/nu15214603)
Supplement: Supplementary file 1 [file nutrients-15-04603-s001.zip › nutrients-2620815-supplementary.pdf]

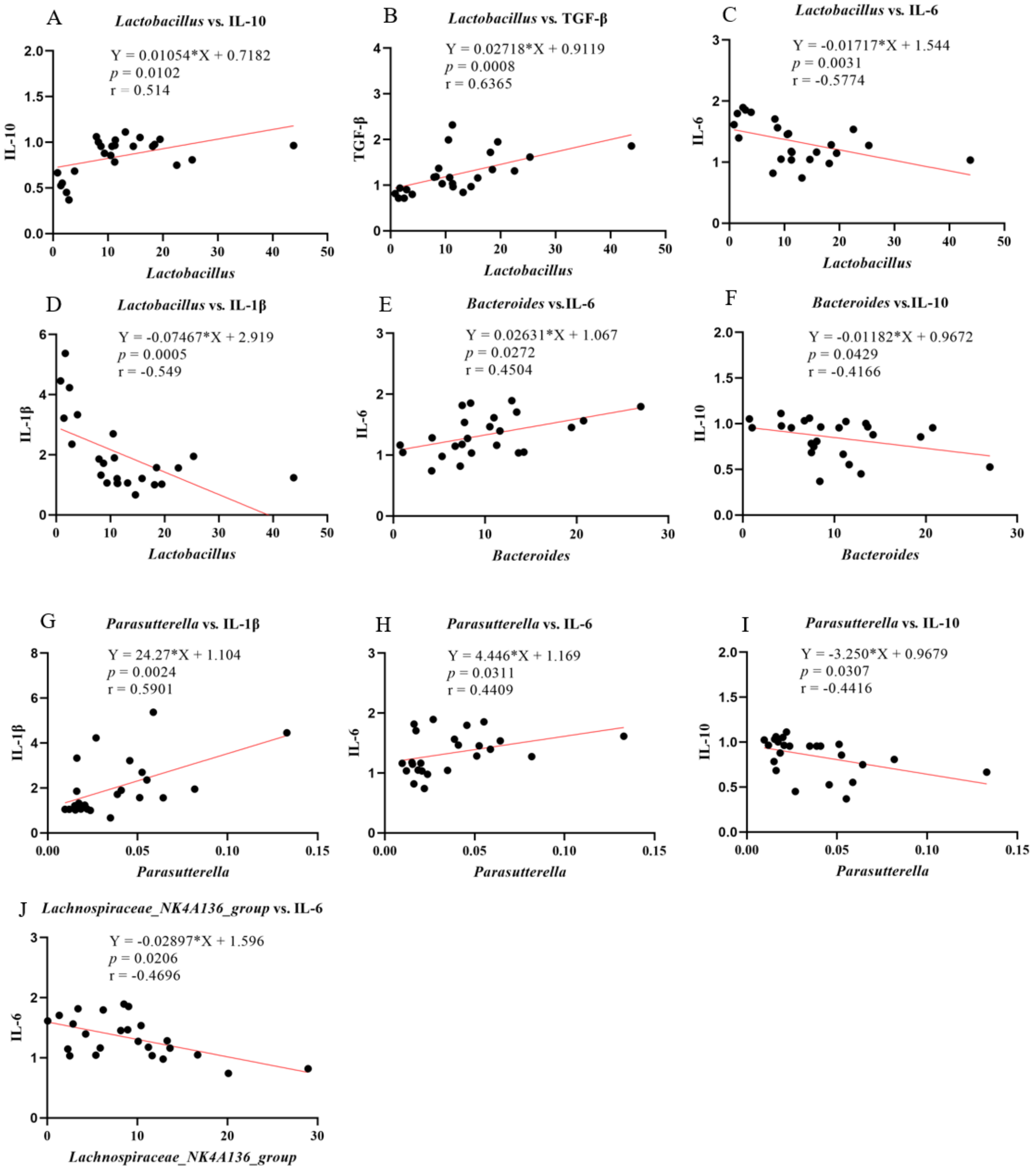

Supplementary Figure S1. Analysis of correlations between cytokine and genus-level intestinal microbes.

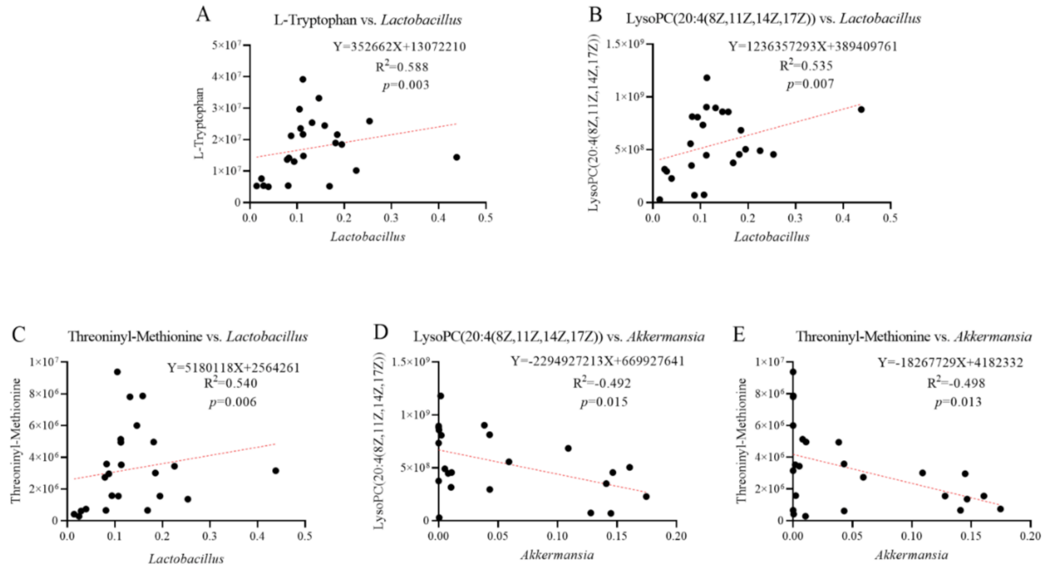

Supplementary Figure S2. Analysis of correlations between differential metabolites and genus-level intestinal microbes.

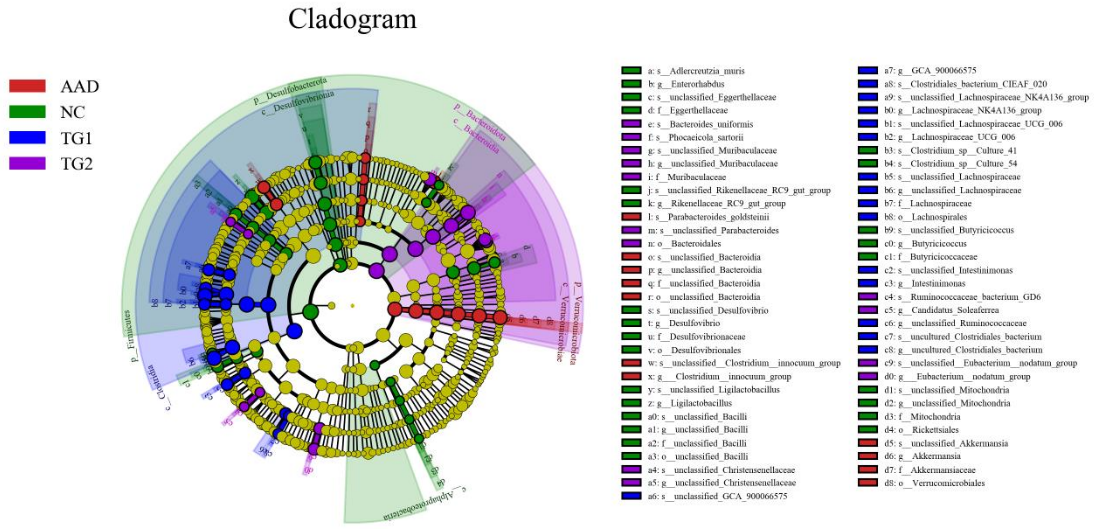

Supplementary Figure S3. LEfSe analysis results of mouse colonic microbiota. The circle radiated from the inside to the outside of the evolutionary branch diagram represents the classification level from gate to species ; each small circle at different classification levels represents a classification at that level. The diameter of the small circle is proportional to the relative abundance. The coloring principle is to uniformly color the species with no significant difference into yellow, and the other different species are colored according to the group with the highest abundance of the species.

Supplementary Table S1. Primer sequences for RT-qPCR detection of colon tissue and amplification of bacterial 16S rRNA genes

Genes/ Region	Forward (5'-3')	Reverse (5'-3')
IL-6	AGCCAGAGTCCTTCAGAGAGA	GCCACTCCTTCTGTGACTCC
IL-1β	AATGCCACCTTTTGACAGTGAT	ATCAGGACAGCCCAGGTCAA
IL-10	CCTGGGTGAGAAGCTGAAGAC	CTTGTAGACACCTTGGTCTTGG
TGF-β	GTGGCTGAACCAAGGAGACG	GTTTGGGGCTGATCCCGTTG
β-actin	GATATCGCTGCGCTGGTCG	CATTCCCACCATCACACCCT
V3-V4	ACTCCTACGGGAGGCAGCA	GGACTACHVGGGTWTCTAAT
